# Universalistic legacy

**DOI:** 10.1007/s00709-022-01757-y

**Published:** 2022-04-02

**Authors:** Peter Nick

**Affiliations:** Botanical Institute, Karlsruhe Institute of Technology, Karlsruhe, Germany

Since the days when the botanist Matthias Schleiden (1838) and his friend, the animal physiologist Theodor Schwann coined the Cell Theory, cell biology has always been a universalistic project. The bold idea that all life is composed of cells and that these cells, despite the diversity of their forms, are equal in their essence, was the forerunner for biology as a science that tries to unify rather than to separate. Other unifying concepts followed—evolutionary theory, genetics, and, eventually, molecular biology, progressively replacing the traditional view that life is composed of diverse but invariant forms. The search for universal rules has since then shaped the central activity of cell biology: comparing. The *ars comparandi* (Nick 2018) requires a continuous oscillation between the particularities of the object in front of our eyes and the generalities deduced from the model we employ to work with this object. To be ready for sometimes daring abstractions is crucial here—this has remained unchanged since the days of Schleiden and Schwann. To put it bluntly, without a certain touch of megalomania it is not possible to do good cell biology.

When the readers open the current issue, they will meet a very fine example for this universalistic spirit – audacious, all-encompassing, and yet dedicated to the details. In his last *opus major*, the late **Thomas Cavalier-Smith** (2021) tries nothing less than re-writing one of the most mysterious chapters of evolution—the genesis of eukaryotes. This work is monumental in several aspects—alone the size of 107 pages breaks all boundaries of conventional research papers. However, to cut this treatise into smaller units would destroy the big picture and the elaborate synthesis of details. Would it make sense to cut the Mona Lisa into puzzle pieces, just to facilitate easier packing? Certainly not. So, it was decided to publish this unique contribution uncut, in its original size. After the first round of peer review, the author passed away before final edits were made, which raised additional questions—how to deal with his work, how to revise a text, and how to handle production and publication processes, whose author cannot be asked any longer? Fortunately, his wife Mrs. Ema Cavalier-Smith/Ms Ema Chao, who had also been his co-author in the past, helped us over the post-acceptance and communication hurdles linked with a posthumous publication. Also, the detailed and meticulous comments of the reviewer were very helpful, such that this unusual publication became possible. If one considers the life and work of the author—for a short, but insightful obituary, see Richards (2021)—it gets clear that this work on the origin of Eukaryotes is a real legacy and, thus, definitely has to be made accessible to the scientific world.

As lever to probe the cloudy origins of eukaryotes, the author makes use of the transition zone, connecting flagella and cilia with the microtubule-organising centriole. This transition zone is far more variable than the rather invariant microtubule architecture in cilia and flagella, and it is also topologically interesting because the microtubule triplets in the centriole have to connect to the central microtubule dyad in the cilium. Interestingly, this transition zone is fairly constant within a taxon and, therefore, represents a valuable cytological trait to infer phylogeny. The core of this transition zone can be built as a dense plate with an asymmetric filament system resembling an acorn leaf. This set-up is predominant across the classical kingdoms and is used to define a new group, the discaria. Deviations of this general scheme are, therefore, of high taxonomical relevance. A recently discovered new group of flagellates, the Malawimonadia, harbours a far more primordial version of this transitional plate and, thus, qualifies as sister to the unknown root of eukaryotes. A second, quite exotic, organism with plastids that lack DNA, *Rhodelphis*, challenges the traditional boundaries between the Chromista and Plantae. By the details of its transition zone, a peculiar nonagonal tube, and a septum around the axoneme of *Rhodelphis*, shared with the hitherto isolated chromist *Picomonas*, this finding shifts the Rhodophyta to the roots, from where the Viridiplantae emerged. The author also comes up with a detailed model, how the transition towards the star-like structure of the Viridiplantae can be imagined. The ultrastructural findings are accompanied by molecular phylogeny, and a thorough knowledge of cellular physiology, for instance, locomotion modes. This comprehensive approach, along with a meticulous analysis of the details in often overlooked and partially newly discovered organisms, certainly contributes to great scientific quality.

However, the main scientific merit of this study is scientific rigour and perseverance. Exceptions are not lazily accepted as the exceptions confirming the rule, but they are taken seriously as thorn in the flesh of an otherwise neatly looking building. As long as there is a thorn in the flesh, the building cannot be considered as finished. It will be an imposition difficult to accept for many, when conventional taxa are overthrown by a new system that will also require to leave classical terminology that has been honoured through decade-long usage. However, it is fruitful to accept the challenge—the new system for the Protists can resolve several inconsistencies and neatly explain details that otherwise would be difficult to understand.

Sure, some of the conclusions are audacious in nature; however, they are always embedded in experimentally testable hypotheses, which renders them fruitful even for those that might not agree with the author. Some of these hypotheses, if fully unfolded, have the potential to shape entire research programme. For instance, he suggests that the cilium of choanoflagellates is homologous to the anterior cilium of the apusozoan biciliate ancestor. At the end of his long journey, the author arrives at 28 conclusions that will advance research and will help us to shed light into the dark past of eukaryotic origins.

Our publication culture favours straightforward stories that are easy to digest and often are dominated by an analytical approach, where molecular details are dissected with great scrutiny. This work here is not easy to digest, and it is dominated by a degree of synthesis that can only be achieved through hard work on a scientific subject through a scientific lifetime. There exist certainly papers easier to handle, requiring less discussions and not demanding difficult decisions. However, there are cases, where it is important to breach conventions for the sake of scientific depth. The work by Cavalier-Smith (2021) is one of these cases.
